# CCL-34, a synthetic toll-like receptor 4 activator, modulates differentiation and maturation of myeloid dendritic cells

**DOI:** 10.18632/oncotarget.7315

**Published:** 2016-02-11

**Authors:** Shu-Ling Fu, Chun-Cheng Lin, Ming-Ling Hsu, Sheng-Hung Liu, Yu-Chuen Huang, Yu-Jen Chen

**Affiliations:** ^1^ Institute of Traditional Medicine, National Yang-Ming University, Taipei, Taiwan; ^2^ Department of Chemistry, National Tsing Hua University, Hsinchu, Taiwan; ^3^ Department of Medical Research, MacKay Memorial Hospital, Taipei, Taiwan; ^4^ School of Chinese Medicine, College of Chinese Medicine, China Medical University, Taichung, Taiwan; ^5^ Department of Radiation Oncology, MacKay Memorial Hospital, Taipei, Taiwan

**Keywords:** CCL-34, TLR4, dendritic cell, differentiation, maturation

## Abstract

CCL-34, a synthetic α-galactosylceramide analog, has been reported as an activator of toll-like receptor 4 (TLR4) in macrophages. TLR4 is highly expressed in dendritic cell (DC) and several TLR4 agonists are known to trigger DC maturation. We herein evaluated the effect of CCL-34 on DC maturation. Human CD14^+^ monocyte-derived immature DC were treated with CCL-34, its inactive structural analog CCL-44, or LPS to assess the DC maturation. CCL-34 induced DC maturation according to their characteristically dendrite-forming morphology, CD83 expression and IL-12p70 production. The allostimulatory activity of DC on proliferation of naive CD4^+^CD45^+^RA^+^ T cells and their secretion of interferon-γ was increased by CCL-34. Phagocytosis, an important function of immature DC, was reduced after CCL-34 treatment. All these effects related to DC maturation were evidently induced by positive control LPS but not by CCL-44 treatment. TLR4 neutralization impaired human DC maturation triggered by CCL-34. The induction of IL-12, a hallmark of DC maturation, by CCL-34 and LPS was only evident in TLR4-competent C3H/HeN, but not in TLR4-defective C3H/HeJ mice. CCL-34 could further elicit the antigen presentation capability in mice inoculated with doxorubicin-treated colorectal cancer cells. In summary, CCL-34 triggers DC maturation via a TLR4-dependent manner, which supports its potential application as an immunostimulator.

## INTRODUCTION

DC are specialized leukocytes that present antigens to naive T cells, thus playing a pivotal role in bridging cell-mediated and humoral immune responses *in vivo* [1]. The ability of DC to stimulate T cells is mainly attributed to their ability to capture antigens, migrate into lymphoid organs, and express high levels of immunostimulatory molecules, such as major histocompatibility complex (MHC) class II, B7.1 (CD80), B7.2 (CD86), and IL-12 [1]. Upon exposure to various microbial and inflammatory products (e.g., lipopolysaccharide [LPS], interleukin-1 [IL-1], tumor necrosis factor-α [TNF-α]), DC matures and migrates into lymphoid tissues to interact with T and B cells [2–5].

Toll-like receptor 4 (TLR4) activation on antigen presenting cells (APCs) can enhance immune responses to antigens and augment the effectiveness of vaccines [6–8]. LPS, the natural TLR4 agonist, causes marked inflammatory responses with major safety consideration for clinical use. Thus, development of novel TLR4 activators to potentiate adaptive immune responses without causing strong inflammation remains an important task [9]. Glycolipid-based TLR4 agonists, such as monophosphoryl lipid A (MPL) and RC-529, have been successfully developed as adjuvant for vaccination. These agents are potent TLR4 activators with better toxicity profile compared with LPS [10].

CCL-34, a synthetic bioactive glycolipid developed previously from our research team, has been reported as a TLR4 activator, promoting macrophage activation and macrophage-mediated cytotoxicity of cancer cells [11], [12]. In a syngeneic bladder cancer cell model, CCL-34 was demonstrated to delay tumor growth via TLR4-dependent activation of immune cells [12]. The tumor sizes in TLR4-defective mice after CCL-34 treatment are close to those in vehicle-treated group, indicating that TLR4 is the main molecular target of CCL-34. Since several TLR4 agonists, such as LPS, are known to be capable of trigger DC maturation, we aimed to evaluate whether CCL34 can promote DC maturation. We used human monocyte-derived immature DC to examine the effect of CCL-34 on their maturation by assessing the morphology, phenotype, cytokine production, stimulation of allogeneic naive T cells. The dependence of CCL-34-induced DC maturation on TLR4 was also demonstrated. Furthermore, the effect of CCL-34 on antigen presentation *in vivo* was analyzed.

## RESULTS

### Morphological changes

**Figure 1:**
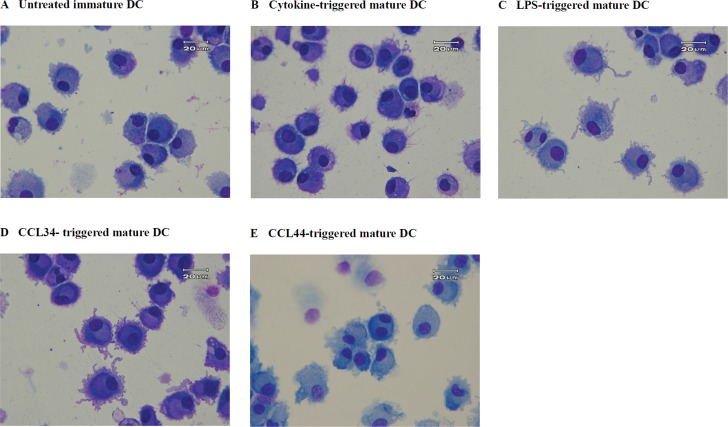
Morphology of monocyte-derived DC (**A**) control immature DC; (**B**) cytokine-triggered mature DC; (**C**) LPS-triggered mature DC; (**D**) CCL-34-triggered mature DC; (**E**) CCL-44-treated DC. Cells were centrifuged onto microscope slides and stained with Liu's solution. Magnification for photograph is 1000×.

Immature DC collected on day 7 before maturation showed round contours without evident dendrites (Figure 1A). The cytokine- and LPS-triggered DC on day 7 had morphological characteristics typical of mature DC, including being non-adherent and having multiple cytoplasmic projections and abundant cytoplasm (Figure 1B–1C). The majority of CCL-34-treated DC manifested similar characteristic features of mature DC, indicating an activity resembling known DC maturation inducers (Figure 1D). By contrast, treatment with CCL-44, an inactive analog of CCL-34, resulted in DC showing less maturation morphology (Figure 1E).

### Effect of CCL-34 on recovery rate of DC

**Figure 2:**
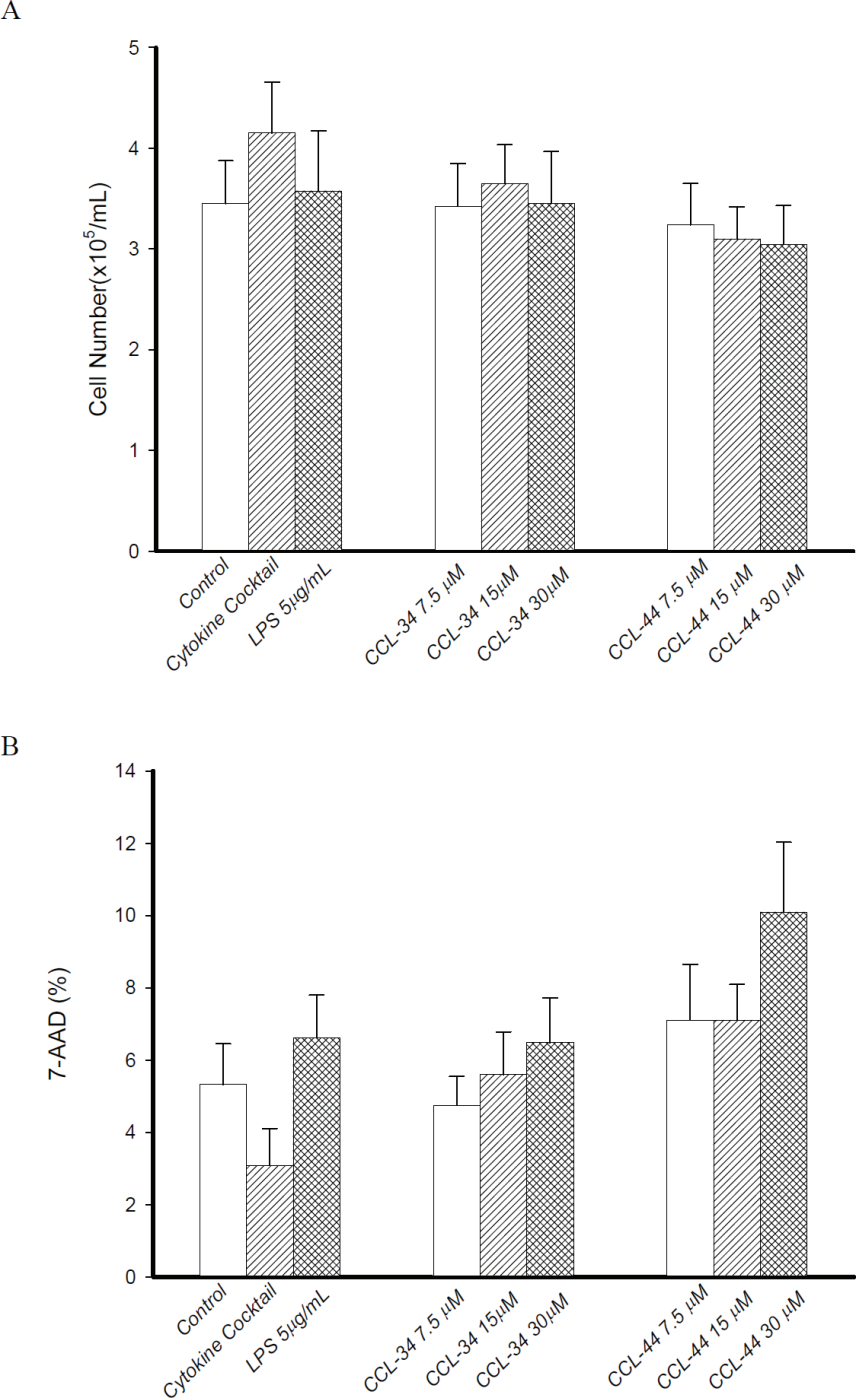
Effect of CCL-34 and CCL-44 on vaibility and cytotoxicity of DC (**A**) Number of viable DC recovered from starting CD14^+^ monocytes was estimated by trypan blue exclusion test. (**B**) Cytotoxicity of CCL-34 and CCL-44 on DC was assessed by 7-AAD staining and flow cytometry. Data from 5 separate experiments by using monocytes from 5 donors are expressed as mean ± SEM.

When CCL-34 was added into culture of immature DC to trigger maturation, there was no significant effect on recovery rate of DC, as measured by trypan blue exclusion test (Figure 2A). Evaluation of cytotoxicity using 7-AAD staining showed that CCL-44, but not CCL-34, induced cell death in an extent greater than LPS (Figure 2B).

### The effect of CCL-34 on DC surface marker expression

**Figure 3:**
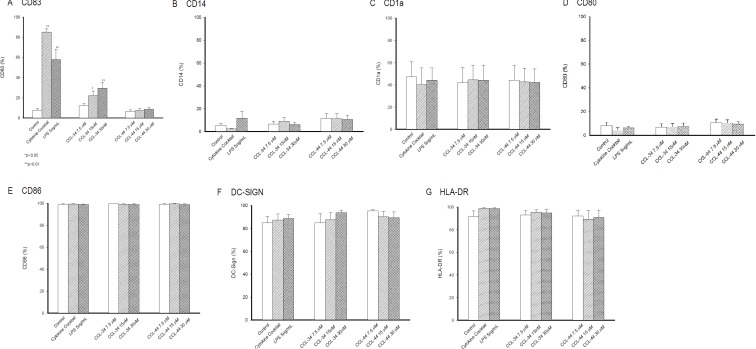
Expression of surface molecules on monocyte-derived DC (**A**) CD83. (**B**) CD14. (**C**) CD1a. (**D**) CD80. (**E**) CD86. (**F**) DC-SIGN. (**G**) HLA-DR. Data from 5 separate experiments by using monocytes from 5 donors are expressed as mean ± SEM.

CCL-34, but not CCL-44, induced markedly expression of CD83 on DC in a dose-dependent manner, indicating a maturation state similar to that triggered by LPS and cytokine cocktail with a lower extent (Figure 3). Since the starting cells were immature DC derived from monocytes, the expression of surface markers related to DC differentiation including CD14, CD1a, co-stimulatory molecules CD80 and CD86, MHC class II molecule HLA-DR, and DC-SIGN were not significantly altered by either CCL-34 or CCL-44 (Figure 3).

### Secretion of IL-12p70 from DC developed in the presence of CCL-34

**Figure 4:**
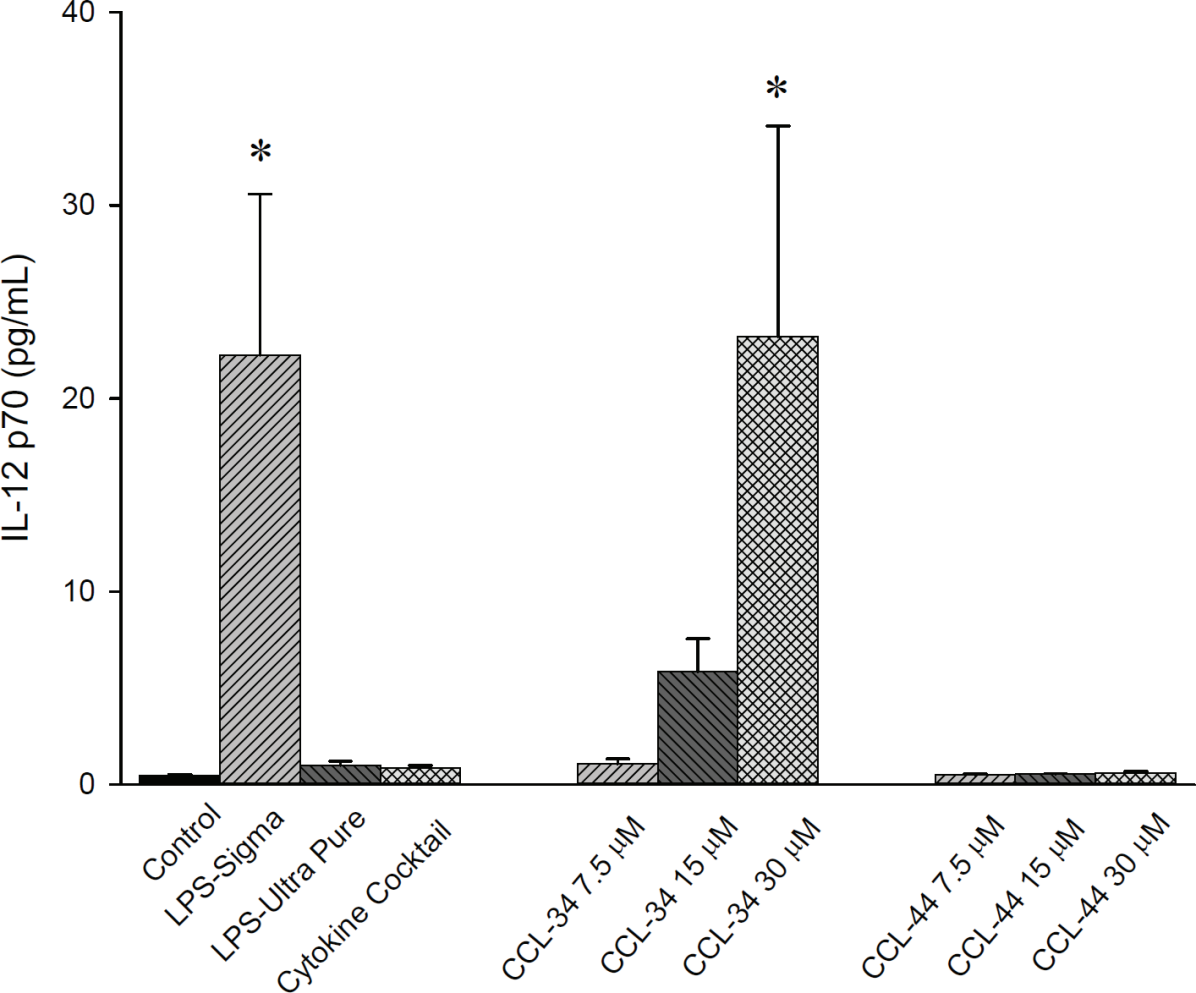
IL-12 production by human myeloid DC Data from 5 to 7 separate experiments are expressed as mean ± SEM. *p-value < 0.05 compared with control group.

As demonstrated in Figure 4, IL-12p70 levels produced by DC in the presence of CCL-34 were dose-dependently increased.

### The effect of CCL-34 on the capacity of DC to stimulate allogeneic naive T cells

**Figure 5:**
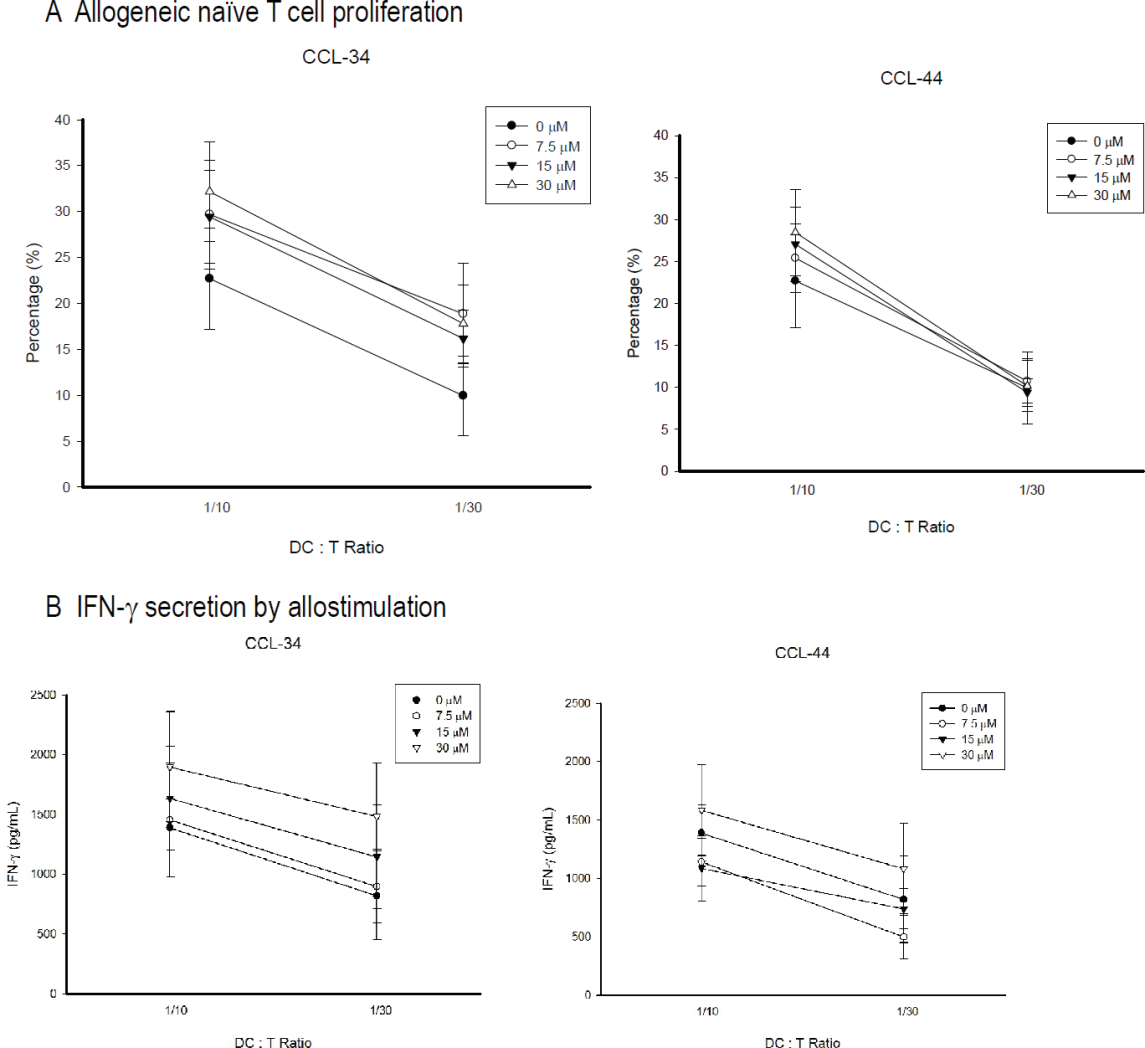
Proliferation and IFN-γ secretion of allogeneic CD4^+^CD45RA^+^ naive T cell stimulated by mature DC The CD4^+^CD45RA^+^ naïve T cells were isolated by using a MiniMACS system with magnetic Abs by a negative selection technique. Monocyte-derived DC were harvested and irradiated (3,000 cGy) with 6 MeV x-ray. Irradiated DC were incubated with 1 × 10^6^ allogeneic naive T cells at ratios of 1:10 or 1:30 for 5 days, after which 10 μM BrdU was added to T cell cultures for 18 h. (**A**) Proliferation. The cells were then collected and the incorporated BrdU was detected using flow cytometry. (**B**) IFN-γ secretion. Data from 5 separate experiments are expressed as mean ± SEM.

CCL-34, but not CCL-44, possessed a trend to augment the allostimulatory activity of DC on stimulating proliferation of naive CD4^+^CD45^+^RA^+^ T cells (Figure 5A). IFN-_γ_ production by these allogeneic naive T cells also had a trend to be enhanced by DC differentiated in the presence of CCL-34, but not CCL-44 (Figure 5B).

### The effect of CCL-34 on phagocytosis of DC

**Figure 6:**
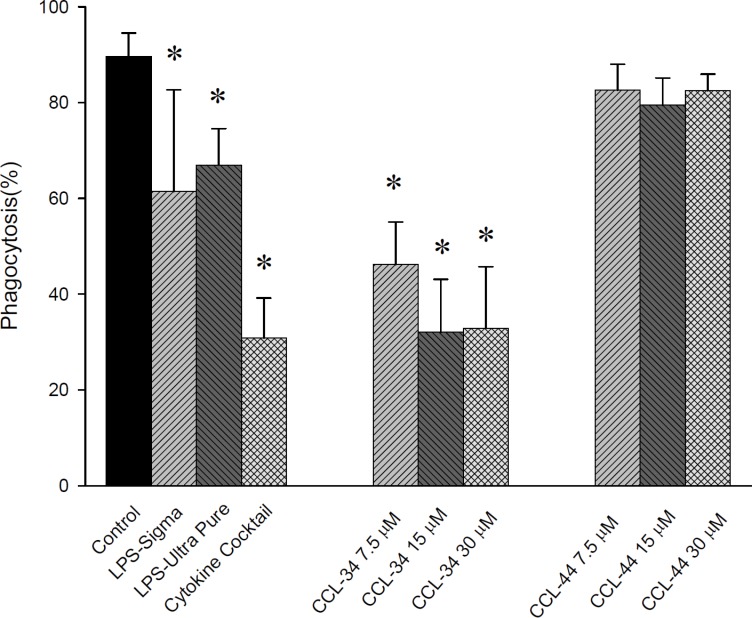
The effect of CCL-34 and CCL-44 on phagocytosis capacity of DC Data from 5 separate experiments by using monocytes from 5 donors are expressed as mean ± SEM. *p-value < 0.05 compared with control group.

Upon stimulation of immature DC toward maturation, the phagocytic capacity is reduced. Our results demonstrated that CCL-34, but not CCL-44, reduced the phagocytosis capacity of DC, suggesting a process toward maturation (Figure 6).

### The effect of CCL-34 on DC is TLR4-dependent

**Figure 7:**
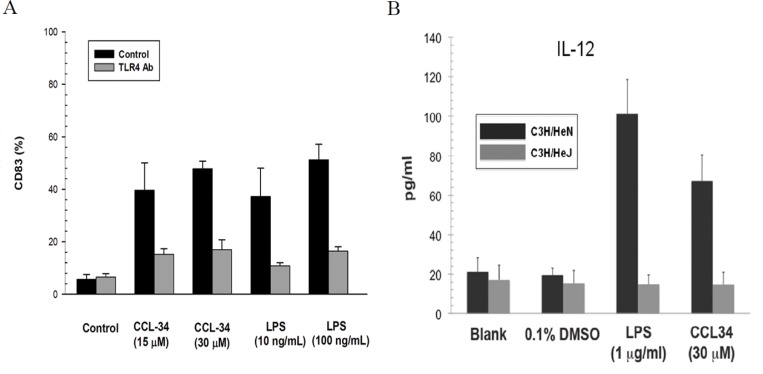
The effect of CCL-34 on DC maturation is TLR4-dependent (**A**) CD83 expression of human DC derived from monocytes of donors with or without TLR-4 antibody blocking. (**B**) IL-12 production by bone marrow-derived immature DC prepared from the TLR4-defective C3H/HeJ and wild-type C3H/HeN mice (5 mice in each group). Data are expressed as mean ± SEM.

Because CCL-34 has been reported as a TLR4 activator in our previous work, we next evaluated whether the effect of CCL-34 on DC maturation is dependent on TLR4. For human myeloid DC, TLR4 neutralizing antibody abolished the maturation effect of CCL-34 and LPS in terms of CD83 expression (Figure 7A). To further elucidate the role of TLR4 on DC maturation triggered by CCL-34, we isolated DC precursors from TLR4-competent C3H/HeN or TLR4-defective C3H/HeJ mice and subjected to differentiation induction toward DC. By measurement of the IL-12 production, it was found that mice DC maturation triggered by CCL-34 and LPS was only evident in TLR4-competent C3H/HeN, but not in TLR4-defective C3H/HeJ mice (Figure 7B). Taken together, the activity of CCL-34 on triggering DC maturation is TLR4-dependent.

### The effect of CCL-34 on DC antigen presentation *in vivo*

**Figure 8:**
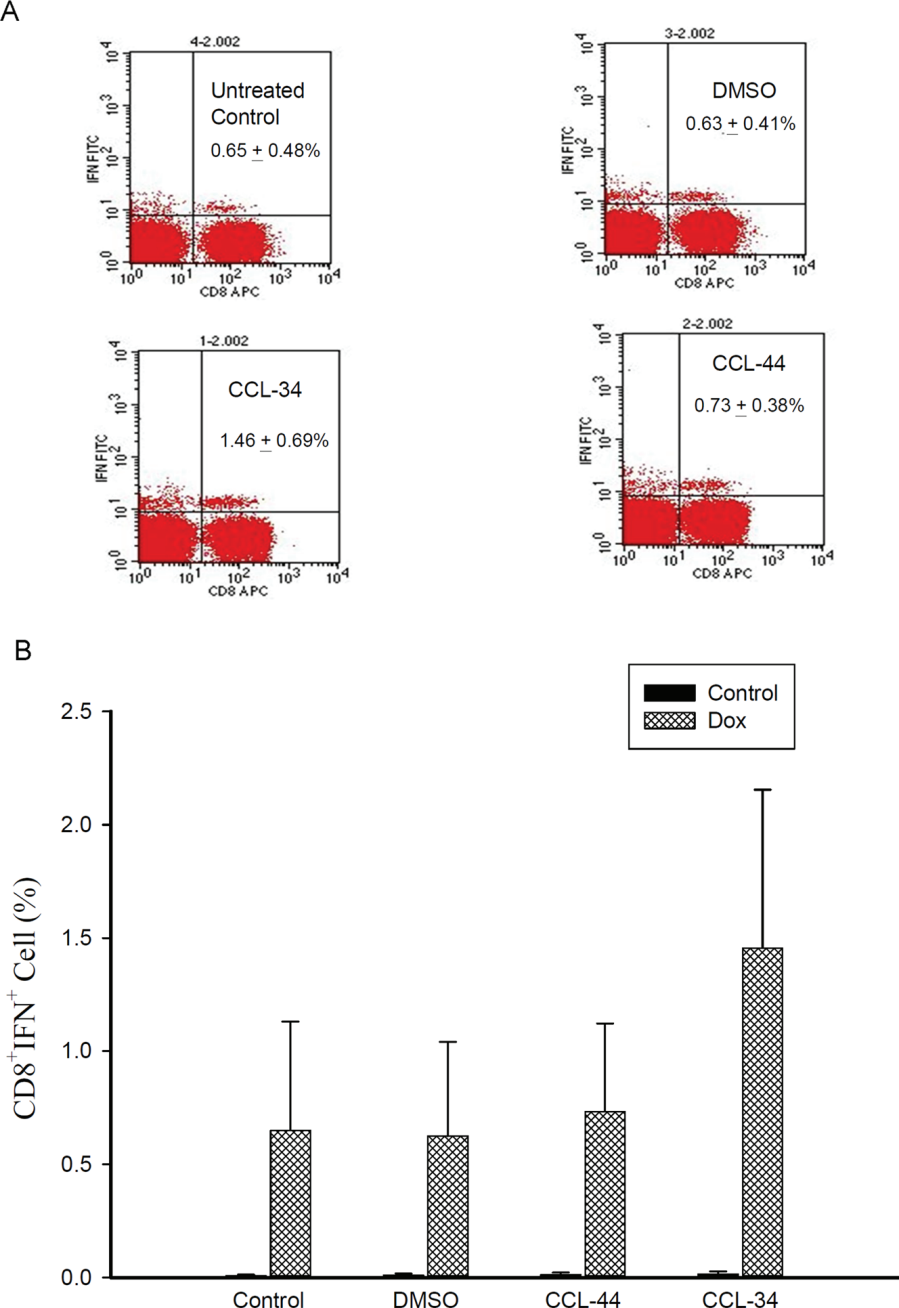
Flow cytometry analysis of IFN-γ–secreting antigen-specific CD8^+^ cell in mice challenged by CT26 cell-associated antigen (**A**) representative flow cytometry data showing the percentage of Ag-specific IFN-γ^+^ CD8^+^ T cells in the tumor draining lymph nodes. (**B**) percentage of IFN-γ^+^ CD8^+^ T cells from each group without (black columns) or with (shaded columns) stimulation by the doxorubicin-treated tumor lysates. Data from 3 separate experiments by using monocytes from 3 donors are expressed as mean ± SEM.

As demonstrated in Figure 8, the amount of antigen-specific CTL in tumor draining lymph nodes of CCL-34-treated mice was greater than those of vehicle and CCL-44, indicating a structure-specific DC immunity *in vivo*.

## DISCUSSION

We here demonstrated that CCL-34, a bioactive synthetic glycolipid developed previously by our team, could trigger DC maturation via a TLR4-dependent manner. The TLR4 activators reported to elicit immunity of DC include glucopyranosyl lipid A [13], *C. nebularis lectin* [14], OM-174 [15], OK-432 [16] and poly-gamma-glutamate [17]. Among currently identified TLR4 activators, CCL-34 is advantageous due to its defined structure and well-established synthesis procedure [18]. Given that CCL34 could activate TLR4 to promote macrophage function [11] and DC maturation, CCL34 may serve as a potential immune modulator for immunotherapy, such as adjuvant for vaccines.

Our previous data demonstrate that CCL-34-activated macrophages are M1-polarized based on their gene expression profiles [12]. The antitumor immunity induced by CCL-34 *in vivo*, including the expression of IFN-γ and IL-12 as well as the leukocyte markers (CD11b, CD11c, CD4 and CD8), further indicated that Th1 adaptive immune responses are activated by CCL-34 [12]. Although the CCL34-induced signaling pathway downstream TLR4 has been elucidated in macrophages [11], [12], [19], it remains not clarified in immature DC. This issue could be addressed further in the disease model using both *in vitro* and *in vivo* experiments.

The role of TLR4 signaling in cancer has been considered a double-edged sword. Activation of TLR4 on immune cells can enhance anti-tumor immunity. However, chronic inflammation related to TLR4 activation remains as a major risk factor in cancer development [20]. Expression of TLR4 and its signaling of tumor cells have been demonstrated to induce the synthesis of soluble immune mediators that could help the tumor to invade the immune attack [21]. In our previous investigation, CCL-34 treatment suppressed tumor growth and benefited the survival in a TLR4-dependent manner [12]. It suggests that CCL-34 may have a potential as an immunotherapeutic agent against cancer *in vivo* without major concern for impairment of immune evasion. Furthermore, CCL-34-treated mice showed induction of IL-12 and elevation of CD11c marker in tumor areas, implying the involvement of dendritic cells in the anticancer activity of CCL-34 *in vivo* [12]. In this study, we provide direct evidence demonstrating that CCL-34 can promote DC maturation, further supporting the idea that activation of dendritic cells by CCL-34 is crucial for its anticancer activity *in vivo*.

Our previous structure-activity relationship analysis of CCL-34 and its analogs revealed that both the sugar moiety and the lengths of the lipid chains are essential for its TLR4-activation activity [11, 18]. In addition, CCL-34 is an optimal TLR4-activating compound among tested structures [12, 18]. It was previously shown that CCL-34 has low toxicity to primary bone marrow cells and its safety has been examined in tumor-bearing mice without major organ toxicity: CCL-34 treatment (i.p. 100 μg/mice, every 3∼4 day in a 21-day treatment) did not significantly decrease body weight, nor affect liver and kidney functions based on serum biochemistry data ([12] and Supplementary data). Based on the safety and the multiple bioactivities we demonstrated in this and previous studies, we think that CCL-34 is a TLR-4 activating immune stimulator meriting further exploration of its potential clinical application.

## MATERIALS AND METHODS

### Generation of human dendritic cells

Human peripheral blood mononuclear cells were obtained from healthy donors using Histopaque (Amersham Pharmacia Biotech, Piscataway, NJ, USA) density gradient centrifugation. Erythrocytes were lysed by treatment with 0.9% ammonium chloride for 3 min at 37°C. Subsequently, CD14^+^ cells were purified by high-gradient magnetic sorting using the miniMACS system with anti-CD14 microbeads (Miltenyi Biotec, Bergisch Bladbach, Germany). After 2 h incubation at 37°C, nonadherent cells were removed and adherent cells were collected. The purity of isolated CD14^+^ monocytes was over 90% on flow cytometric analysis. Immature DC were generated from CD14^+^ monocytes by culture in RPMI 1640 medium supplemented with 10% fetal calf serum, 100 ng/mL granulocyte-macrophage colony-stimulating factor (GM-CSF) (Schering-Plough, Munich, Germany) and 50 ng/mL IL-4 (R & D Systems, Minneapolis, MN, USA) every 3 days for 6 days in a humidified 5% CO_2_ incubator. To trigger maturation of DC, immature DC were incubated with LPS, CCL-34 (0, 7.5, 15, 30 mM), and combination of pro-inflammatory cytokines including 5 ng/mL TNF-α, 5 ng/mL IL-1β, 15 ng/mL IL-6 (R & D Systems, Minneapolis, MN) and 1 μg/mL prostaglandin E2 (PGE2) (Sigma-Aldrich, St. Louis, MO, USA) for a further 24 h. To examine the effect of LPS as a positive control, we tested two kinds of LPS to select an appropriate stimulator for DC maturation. As shown in Figure 4, LPS, but not LPS-Ultra pure, could stimulate IL-12p70 production from DC. Although not statistically significant, the mean phagocytosis percentage of DC in LPS group was smaller than in LPS-Ultra pure group, implicating a more potent maturation induction by LPS. According to these data, we selected LPS as positive control thereafter. It has been shown that monocyte-conditioned medium containing a combination of pro-inflammatory cytokines (i.e. TNF-α, IL-1, IL-6 and PGE2) triggers efficient DC maturation. Thus, we used a cocktail of these cytokines with defined concentrations that had also been applied by other researchers for this purpose. CCL-34 and CCL-44 were synthesized by Dr. Chun-Cheng Lin's laboratory at National Tsing Hua University (Hsinchu, Taiwan) and dissolved in DMSO. The structure and synthetic scheme of CCL-34 and CCL-44 were described in [11]. The possibility of LPS contamination in both compounds has been excluded [11].

### Cell viability

DCs were harvested on day 8 and the numbers of viable cells were counted using the trypan blue dye exclusion test. The recovery rate of DC was estimated by dividing the number of harvested DC by the total number of sorted CD14^+^ monocytes. The amount of dead cells was estimated by using 7-AAD staining and flow cytometry analysis.

### Surface antigen expression on DC by flow cytometric analysis

Dual-color immunolabelling was performed using fluorescein isothiocyanate (FITC)- and phycoerythrin (PE)-conjugated monoclonal antibodies (mAbs). The mouse anti-human mAbs IgG1:FITC/mouse IgG1:PE, and appropriate isotype controls were purchased from Serotec (Oxford, U.K.) and used for DC characterization as follows: anti-CD14 (for IgG-FITC), anti-CD1a-PE, anti-CD80-PE, anti-CD86-PE, anti-CD83-PE, anti-HLA-DR-PE and anti-DC-SIGN-PE. Cells were incubated with saturating concentrations of PE-conjugated mAbs or primary mAbs followed by IgG-FITC at 4°C for 30 min. After washing twice with PBS, 1 × 10^6^ cells were applied to a FACS caliber flow cytometer (BD Biosciences, San Jose, CA). Data were collected and analyzed using CellQuest Software (BD Biosciences).

### Phagocytosis assay

A quantitative measurement for the phagocytic capacity of DCs was performed by allowing the cells to engulf large (2 μm in diameter) immunofluorescent latex beads (FITC-conjugated dextran-modified latex bead, Sigma). Cells were harvested and allowed to phagocytose the beads for one hour at 4 or 37°C. The cells were washed for three times and then subjected to flow cytometer analysis. The quantity of phagocytosis by DCs is represented as percentages of FITC-positive cells. Data were normalized to phagocytosis obtained with untreated DCs at 4°C.

### Morphological observation

Harvested DC were centrifuged onto microscope slides by a Cytospin^2^ centrifuge (Shandon Inc, Pittsburgh, PA), stained with Liu's solution, and observed under light microscopy (Olympus, Tokyo, Japan). Photographs were taken with a digital camera equipped on microscopy.

### Allogeneic naive T cell proliferation

To purify CD4^+^CD45RA^+^ T cells, nonadherent cells from culture of isolated mononuclear cells were used. Naive T cells were enriched with a CD4^+^CD45RA^+^ T cell isolation kit (Miltenyi Biotec) using a MiniMACS system with magnetic Abs by a negative selection technique. Monocyte-derived DC were harvested and irradiated (3,000 cGy) with 6 MeV X-rays generated by a linear accelerator (Clinac^®^ 1800, Varian Associates, Inc., CA) at a dose of 4.0 Gy/min in a single fraction. Full electron equilibrium was ensured for each fraction by a parallel plate PR-60C ionization chamber (Capintel, Inc., Ramsey, NJ, USA). Thirty Gray-irradiated DC were incubated with 1 × 10^6^ allogeneic naive T cells at ratios of 1:10 or 1:20 for 5 days, after which 10 μM 5-bromo-2-deoxyuridine (BrdU) was added to T cell cultures for 18 h. The cells were then collected and the incorporated BrdU was detected using flow cytometry.

### Detection of cytokines produced by DC and stimulated allogeneic naive T cells

For human DC, the levels of IL-12_p70_ and IL-10 in the DC supernatant as well as interferon-γ (IFN-γ) in the stimulated allogeneic T-cell supernatant were measured using ELISA kits purchased from R & D Systems according to the manufacturer's instructions. The detection limits for cytokines were 5.0–8.0 pg/mL. For mouse DC, the IL-12p70 secreted by DC was measured using ELISA kit purchased from R & D systems, Vienna, Austria).

### IL-12 produced by maturated DC from TLR4-defective and wild-type mice

To clarify whether CCL-34 modulate DC maturation via a TLR4-dependent manner, bone marrow-derived immature DC prepared from the TLR4-defective C3H/HeJ and wild-type C3H/HeN mice were isolated and cultured as described previously [22]. The growth medium contains 20 ng/ml rmGM-CSF and 20 ng/ml rmIL-4 for promoting DC differentiation. After incubation for 3 days, the suspended cells were removed and fresh growth medium was added to the adherent cells, which were maintained for an additional 3 days. Then suspension and loosely adherent cells were harvested for subsequent treatment with vehicle, CCL-34 or LPS for 24 hr, and assessed for IL-12 production using ELISA assays as previously described.

### DC antigen presentation *in vivo* and analysis of antigen-specific CTL

For DC antigen presentation *in vivo*, immunocompetent Balb/c mice were treated by intraperitoneal injection of vehicle, CCL-34 or CCL-44 for 3 consecutive weekly dosage of 5 mg/kg. Mice were subcutaneously injected with doxorubicin (5 μM for 24 h)-treated syngeneic colorectal cancer CT26 cells. After one week, the mice were implanted with live CT26 cells at flank and the draining popliteal lymph nodes were removed after tumor grew to greater than 3 mm in diameter. Single cell suspension prepared from these draining lymph nodes were further challenged by lysates of untreated and doxorubicin-treated CT26 cells for 24 h and then subjected to analysis for antigen-specific CTL. Toward this end, the expression of surface molecule CD8 and the staining for intracellular IFN-γ was analyzed by flow cytometry.

### Statistical analysis

The results were expressed as mean ± standard error of mean (SEM). Comparison in each experiment was performed using one-way analysis of variance followed by Dunnett's test, and a *p* value of less than 0.05 was considered statistically significant.
